# Enteric Viruses and Inflammatory Bowel Disease

**DOI:** 10.3390/v13010104

**Published:** 2021-01-13

**Authors:** Georges Tarris, Alexis de Rougemont, Maëva Charkaoui, Christophe Michiels, Laurent Martin, Gaël Belliot

**Affiliations:** 1 Department of Pathology, University Hospital of Dijon, F 21000 Dijon, France; georges.tarris@chu-dijon.fr (G.T.); laurent.martin@chu-dijon.fr (L.M.); 2 National Reference Centre for Gastroenteritis Viruses, Laboratory of Virology, University Hospital of Dijon, F 21000 Dijon, France; alexis.de-rougemont@u-bourgogne.fr; 3 Department of Hepatogastroenterology, University Hospital of Dijon, F 21000 Dijon, France; maeva.charkaoui@chu-dijon.fr (M.C.); christophe.michiels@chu-dijon.fr (C.M.)

**Keywords:** norovirus, rotavirus, astrovirus, sapovirus, adenovirus, aichi virus, Crohn’s disease, ulcerative colitis, inflammatory bowel disease

## Abstract

Inflammatory bowel diseases (IBD), including ulcerative colitis (UC) and Crohn’s disease (CD), is a multifactorial disease in which dietary, genetic, immunological, and microbial factors are at play. The role of enteric viruses in IBD remains only partially explored. To date, epidemiological studies have not fully described the role of enteric viruses in inflammatory flare-ups, especially that of human noroviruses and rotaviruses, which are the main causative agents of viral gastroenteritis. Genome-wide association studies have demonstrated the association between IBD, polymorphisms of the *FUT2* and *FUT3* genes (which drive the synthesis of histo-blood group antigens), and ligands for norovirus and rotavirus in the intestine. The role of autophagy in defensin-deficient Paneth cells and the perturbations of cytokine secretion in T-helper 1 and T-helper 17 inflammatory pathways following enteric virus infections have been demonstrated as well. Enteric virus interactions with commensal bacteria could play a significant role in the modulation of enteric virus infections in IBD. Based on the currently incomplete knowledge of the complex phenomena underlying IBD pathogenesis, future studies using multi-sampling and data integration combined with new techniques such as human intestinal enteroids could help to decipher the role of enteric viruses in IBD.

## 1. Introduction

Inflammatory bowel disease (IBD), which includes ulcerative colitis (UC) and Crohn’s disease (CD), is an important public health issue with an estimated 6.8 million cases worldwide and prevalence rates ranging from 79.5 to 84.3 per 100,000 individuals [1]. Both CD and UC are characterized by chronic mucosal inflammation, with CD affecting proximal to distal segments of the gastrointestinal tract (stomach, small intestine, large intestine) and UC affecting only the distal segments (colon and rectum) [2]. The pathological features of CD and UC are different. In CD, there is transmural inflammation involving the full thickness of the bowel, with lymphoplasmacytic aggregates, epithelioid granulomas, mucosal edema, scarring fibrosis, and neural thickening of tissues. In UC, histopathology usually shows patchy mucosal ulcerations, mucosal edema, hemorrhagic foci of the lamina propria, and cryptic damage [2]. Paneth cell metaplasia has been described in the distal colon in both conditions [3]. Altogether, IBD involves microbial, environmental, dietary, immunological, and genetic factors, though the mechanisms underlying intestinal inflammation remain debated [4,5]. While the effect of cytomegalovirus (CMV) infection on IBD has been confirmed, the role of enteric viruses in inflammatory bursts of IBD, pathogenesis, and exacerbation of disease symptoms is controversial [6,7]. Despite recent data suggesting that there is an increased risk of serious viral infections in IBD patients, especially in those undergoing prolonged IBD therapy, the risk factors and predictors of enteric virus infections have not been clearly identified in these individuals [8]. Enteric viruses include the *Picornaviridae* (enteroviruses, polioviruses, coxsackie viruses, echoviruses, and hepatitis A virus), *Adenoviridae* (adenovirus), *Caliciviridae* (norovirus and sapovirus), *Astroviridae* (astrovirus), and *Reoviridae* (rotaviruses) families. Their role in IBD pathogenesis remains largely unknown. Here, we review various aspects of enteric virus infections in IBD, including epidemiology, virus–host interactions, gut microbiota, inflammatory pathways, virome studies, and therapeutic approaches.

## 2. Epidemiology of Enteric Viruses in IBD

Few epidemiological studies have focused on the prevalence of enteric viruses in IBD (Table 1), and the conclusions of these studies can be contradictory because of the techniques used for virus detection (RT-PCR or rapid antigenic test) or the small size of the cohorts. The first epidemiological study was conducted in 1982 by Gebhard et al. [9]. The study aimed to detect norovirus and rotavirus antigens in stool samples from IBD patients in combination with serological analysis. For 14 months, 77 patients were followed, during which 65 flare-ups were observed in 54 patients. A total of 266 stool samples were tested, of which 55 were obtained during disease exacerbation. The study was not able to make a clear association between IBD relapse and enteric virus infections. However, the authors concluded that five out of eight patients with serological evidence of enteric virus infection showed symptoms of exacerbation during a 3-month period (*p* < 0.01) [9]. Another epidemiological study by Khan et al. compared the medical records of a pediatric emergency department and norovirus-positive stool specimens (*n* = 2666 cases) over a 10-month period [10]. Nine patients (1 CD and 8 UC) with bloody diarrhea linked to IBD relapse had at least one positive stool for norovirus antigen using the rapid test. Considering that the cohort included 23 CD and 14 UC patients, the study showed a significant association between UC relapse and norovirus presence in stool (*p* < 0.01) [10]. Three of the norovirus-positive IBD patients were coinfected with either *Clostridium difficile*, adenovirus, or rotavirus [10]. Conversely, a prospective screening of 50 stool specimens for norovirus, rotavirus, astrovirus, and sapovirus in a Swedish pediatric IBD cohort during routine endoscopy procedures showed no prevalence of enteric viruses during IBD flare-ups; however, a very low prevalence of GII noroviruses or sapoviruses was found in non-IBD patients in which IBD was ruled out by endoscopy [11]. Another prospective study in a Dutch cohort aimed to detect enteric viruses in stool samples from 286 IBD patients during their 1-year trimestral follow-up. The results suggested a low prevalence of enteric viruses in both baseline and active IBD, and no epidemiological link could be established between disease activity and enteric virus infections [12]. The largest and most recent study showed a clear association between enteric viruses and IBD flare-ups using data-crossing from 9403 patients in whom 13,231 stool tests were performed using a gastrointestinal pathogen PCR panel over a 2-year period. Here, Axelrad et al. compared 577 IBD patients (277 CD and 300 UC) to 8826 controls and demonstrated the greater prevalence of enteric infections (such as norovirus; *p* < 0.001) as well as *Enteroinvasive Escherichia coli* (EIEC) in both CD and UC patients during IBD flare-ups [13,14].

## 3. Histo-Blood Group Antigens and Virus–Tissue Interactions in Patients with IBD

**Figure 1:**
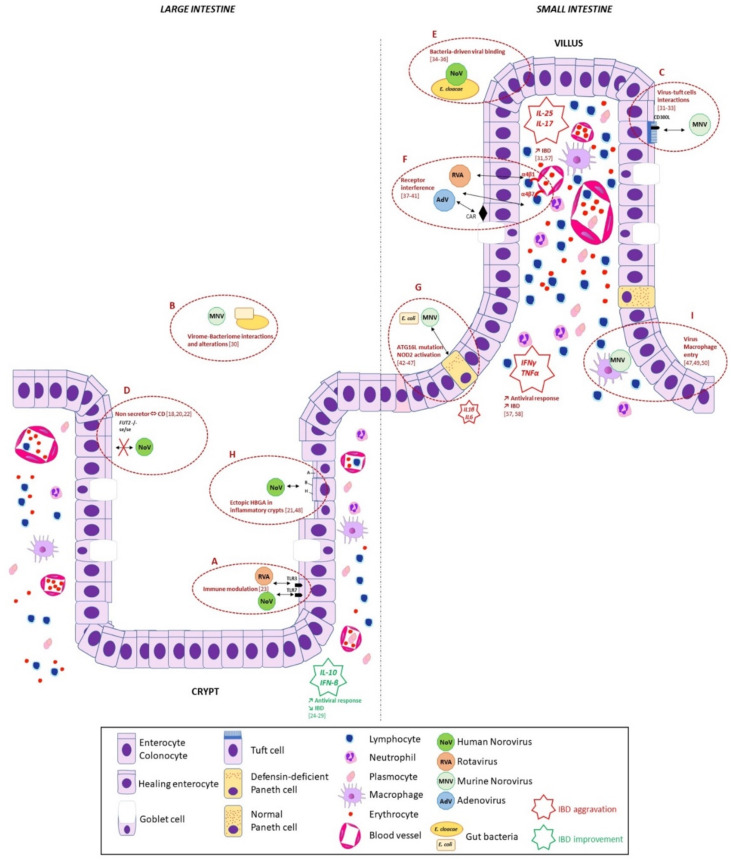
Summary of potential mechanisms underlying enteric virus interactions during the course of inflammatory bowel disease (IBD). Inflammatory pathways involved in IBD pathogenesis are shown in red and green stars. (**A**) Enteric viruses, such as rotavirus and norovirus, may modulate the immune response during IBD and improve the symptoms of IBD through the increased secretion of interferon β (IFN β) [23]. The protective role of interleukin (IL)-10 was confirmed in IL-10^-/-^ murine models showing enterocolitis after exposition to enteric viruses [24,25,26,27,28,29]. (**B**) Interactions of the gut bacteriome and virome could also explain IBD pathogenesis, for instance, an asymptomatic murine norovirus (MNV) infection could replace the effects of beneficial commensal bacteria [30]. (**C**) Recent scientific data revealed that norovirus may bind to tuft cells, which would then play an important role in the IL-25/IL-17 inflammatory pathway, as seen during IBD [31,32,33]. (**D**) The absence of histo-blood group antigen (HBGA) synthesis in non-secretor subjects carrying polymorphisms of the *FUT2* and *FUT3* genes could be linked to altered virus–bacteria–cell interactions and involved in the pathogenesis of IBD [18,20,22]. (**E**) Interactions between enteric viruses and HBGA-like molecules at the surface of commensal or pathogenic bacteria modulated by an “IBD-shaped” microbiota could explain the variations in susceptibility to enteric viruses during IBD [34,35,36]. (**F**) Binding of enteric viruses to critical receptors involved in leukocyte adhesion and cytokine secretion could also be part of IBD pathogenesis, as exemplified by rotavirus and adenovirus interactions with integrins, or activation of the Coxsackie–Adenovirus Receptor (CAR) [37,38,39,40,41]. (**G**) Specific interactions between enteric viruses (MNV) and defensin-deficient Paneth cells in patients carrying polymorphisms of the *ATG16L* gene could lead to the activation of the *NOD2* gene and upregulate T-helper 1 (Th1) or T-helper 17 (Th17) inflammatory pathways, as seen in IBD [42,43,44,45,46,47]. (**H**) Ectopic expression of HBGA in intestinal epithelium undergoing alterations induced by inflammation may modulate the binding potency of enteric viruses [21,48]. (**I**) Enteric virus entry in macrophages stimulates tumor necrosis factor alpha (TNF-α) and interferon gamma (IFNγ) secretion, which are involved in IBD pro-inflammatory pathways [47,49,50].

Enteric viruses, noroviruses, and rotaviruses use histo-blood group antigens (HBGAs) as natural ligands with a significant duodenal tropism [15]. The expression of HBGAs is mainly due to the expression of α1,2-fucosyltransferase (FUT2), catalyzing the addition of a fucose with an alpha-1,2 linkage to disaccharide precursors at the surface of enterocytes [15,16]. While several *FUT2* mutations have been reported, the G428A mutation is the most commonly described in European and Mediterranean populations, and it results in a “non-secretor phenotype” and the absence of expression of ABO antigens at the surface of secretor-dependent tissues (i.e., tissues in which the expression of ABO blood group antigens is non-constitutive and conditioned by the *FUT2* gene). The synthesis of Lewis antigens is consecutive to H_1_ chain expression and is driven by the *FUT3* gene, which encodes α1,3-fucosyltransferase (FUT3). The enzyme adds a fucose with an alpha-1,3 linkage to an H_1_ precursor to produce Lewis b (Le^b^, major form) and Lewis y (Le^y^, minor form) antigens in the presence of an active *FUT2* gene. In the absence of active *FUT2*, *FUT3* adds a fucose to a disaccharide precursor to produce Lewis a (Le^a^, major form) and Lewis x (Le^x^, minor form) antigens [17]. A genome-wide association study (GWAS) conducted in Chinese patients diagnosed with UC demonstrated higher frequencies of mutant *FUT3* gene in UC patients compared to controls [18]. Higher frequencies of *FUT3* mutants were also observed in patients with distal colitis, along with an increased expression of the Lewis a antigen in the inflamed colon [18]. Other GWAS have demonstrated that multiple single nucleotide polymorphisms (SNPs) of the *FUT2* gene were associated with CD pathogenesis due to alterations of HBGA expression in the gut [19,20]. Unlike ileal CD, a Japanese study has documented the weak occurrence of relapses of colonic CD in non-secretor patients, which was related to the ectopic expression of secretor-dependent blood group antigens in inflammatory mucosa (Figure 1) [21]. Furthermore, Rausch et al. compared the bacterial composition in patients with CD and either a secretor or non-secretor phenotype. Secretor and non-secretor individuals had significant differences in their bacterial composition. Variations in HBGA composition changed the capacity for bacterial attachment and nutrition, with greater concentrations of *Lachnospiraceae* and decreased concentrations of *Faecalibacterium* or *Lactobacillus* spp. That being said, no correlation was established with enteric viruses [22].

There is a clear interaction between noroviruses and intestinal tissues in pathologic conditions. For instance, greater expression of the sialylated Lewis x antigen (CD15s) has been described in Portuguese patients suffering from chronic gastritis and carrying the Cag-A positive *Helicobacter pylori* strain. However, there was no clear explanation for the phenomena underlying pathologic alterations of the glycosylation processes during inflammation, whether due to the display of the *H. pylori* strain or to pathologic alterations induced by inflammatory processes (Figure 1) [48]. The exact role of enteric viruses and their interactions with intestinal cells in the context of IBD is relatively unknown. However, there is increasing evidence of their implications in the dysregulation of inflammatory pathways and their complex interactions with bacterial and yeast organisms.

## 4. Enteric Virus Interactions with Bacteria in IBD

The interactions between enteric viruses and bacteria are complex and remain poorly explored: viral enteric infections could be promoted by intestinal eukaryotic bacteria, and bacterial infections could be promoted by enteric viruses [51]. For example, the selective interaction of noroviruses with bacteria expressing HBGA-like molecules, such as the *Enterobacter cloacae* SENG-6 strain, could inhibit norovirus binding on intestinal cells or, on the contrary, enhance norovirus infection (Figure 1) [34,35,36]. The decrease in *Enterobacter* sp. during severe IBD could also increase the probability of norovirus infection in the colonic mucosa [52]. Recent scientific literature has confirmed that the occurrence of IBD or IBD-like symptoms in mice, induced by murine noroviruses (MNV), might be highly dependent upon the composition of the murine gut microbiota without altering its composition [53,54]. Of course, murine models might tend to differ from human models, especially in complex multifactorial diseases like IBD. Microbiome studies have shown that human norovirus infections could disrupt the gut microbiota, resulting in a reduced number of bacteroidetes and increased proteobacteria, especially non-virulent *E. coli* strains [55,56]. Murine models further demonstrated that MNV could even shape the microbiota and replace the function of commensal bacteria in germ-free mice (Figure 1) [30]. Further studies will be required to detail the mechanisms underlying virome and bacteriome interactions in IBD patients and their role in the dysregulation of inflammatory pathways underlying IBD relapse.

## 5. Enteric Viruses and Inflammatory Pathways in IBD

The cellular mechanisms that underlie the activation of mucosal inflammation in IBD have largely been explored in recent decades, including the recruitment of inflammatory cells in the mucosa and the secretion of proinflammatory cytokines. The role of interleukin-17 (IL-17) and interferon gamma (IFNγ) in the T-helper 1 (Th1) and T-helper 17 (Th17) lymphocytic response is known to worsen the progression of CD and UC (Figure 1) [31,57]. Tumor necrosis factor alpha (TNF-α) is also known to increase proinflammatory interleukins at a mucosal level, and therefore increase mucosal inflammation [58]. By contrast, protective molecules like interleukin-10 and tumor growth factor beta (TGF-β) protect the mucosa from colitis and stimulate epithelial repair and ulcer healing [24,59]. Noroviruses are known to replicate in macrophages with the inhibition of replication mediated by IFNγ [49,50]. Interestingly, recent studies have shown that rotaviruses bind to α4β1 integrins in MA104 and Caco-2 cell lines, whose integrins are involved in leukocyte adhesion and hematopoiesis [38,39,40]. Adenoviruses also use α4β1 and α4β7 integrins for binding in HT29 cells (Figure 1) [37]. Further investigations are needed to better understand the effects of enteric viruses in lymphocyte homing and their role in mucosal inflammation.

Current scientific data about the mechanisms underlying the putative role of enteric viruses in mucosal inflammation come mainly from murine models for practical, ethical, and financial reasons. Interestingly, enteric virus infections, including poliovirus, norovirus, and rotavirus, appear to be promoted by interactions with commensal bacteria through the action of bacterial lipopolysaccharides in murine models and cultured human B cells [60,61,62,63,64]. MNV could also disrupt the epithelial barrier and induce epithelial injury in IL-10-deficient mice, who are known to develop chronic enterocolitis, though without a clear understanding of the repercussions on gut microbiota [25,26]. Further studies conducted in homozygous IL-10-deficient mice showed that MNV had no effect on the course of *H. pylori*-induced IBD, but it did have immunomodulatory effects on *H. bilis*-driven IBD, showing high secretion of IFNγ by polyclonal T cells [27,28,29].

As for human norovirus, norovirus challenge studies have revealed elevated levels of Th1 lymphocytic cytokines such as TNFα, IL-8, and IL-10 [65]. More precisely, IFNα and IFNβ, but also IFNλ, could induce an antiviral gene expression cascade following norovirus or rotavirus infections (Figure 1) [66,67,68]. The role of enteric virus infections in the dysregulation of the interferon pathway during IBD remains unclear, especially because many patients undergo anti-TNF therapy, which in turn may downregulate the interferon antiviral response and alter the course of intestinal epithelial repair [69,70].

Conversely, the protective role of enteric viruses in the course of IBD is shown by other mechanisms: enteric viruses could alleviate intestinal inflammation via the production of IFNβ, mediated by toll-like receptors (TLR) 3 and 7 (Figure 1) [23].

Concerning the IL-17 pathway (Th2 lymphocyte activation), recent studies have incriminated tuft cells, which are an immune mediator that plays a role in MNV pathogenesis. MNV could bind to tuft cells via the CD300L receptor and induce proliferation, which in return could stimulate the synthesis of interleukin 25 (IL-25) and therefore stimulate intestinal modeling and inflammation during IBD (Figure 1) [32,33].

## 6. Enteric Viruses and Autophagy in IBD

GWASs have helped to decipher the role of several autophagy genes involved in the pathogenesis of IBD [19]. The *NOD2* gene encodes for a pattern recognition receptor (PRR) that recruits ATG16L at the bacterial infection site [44]. It is well known that *NOD2* gene mutations induce CD and UC through exaggerated activation of NF-κB in monocytes following bacterial stimulation [45,46]. In murine models, aberrant packaging of lysosomal granules in Paneth cells of the intestine linked to *ATG16L1^HM^* mutations is triggered by MNV infection, which then leads to altered immune function in the mucosal barrier, reduced autophagy, and an increased risk of IBD (Figure 1) [42]. More specifically, in the context of CD, *ATG16L1^HM^* polymorphisms may modulate proinflammatory responses upon *NOD2* activation, inducing IL-1β and IL-6 release by peripheral blood mononuclear cells [43]. Moreover, MNV specifically activates *NOD1* and *NOD2* signaling pathways following macrophage entry, stimulating TNF-α production secondary to infection with pathogenic *E. coli* [47].

## 7. Enteric Virome Studies in IBD

It has recently been hypothesized that the mammalian intestinal virome could be related to intestinal homeostasis in relationship to eukaryotes and prokaryotes. This virome may thus contribute to disease pathogenesis through microbial lysis, epithelial cell infection, or immune activation following translocation through epithelial cells [71]. Next-generation sequencing (NGS) has recently been used to study the enteric virome. However, these studies show considerable discrepancies in viral identification and significant interindividual variability [72,73]. In captive rhesus macaques suffering from idiopathic chronic diarrhea (ICD), a disease similar to UC, virome studies revealed an increased prevalence of *Picornaviridae* (including poliovirus and enterovirus) compared to healthy controls [74]. A vast expansion of *Caudovirales* bacteriophages has been shown in the gut virome of human IBD patients, without any further characterization of pathogenic enteric viruses [75,76]. Additionally, high levels of *Anelloviridae* have been found in the stool of patients undergoing long-term immunosuppressive therapy [77]. A study conducted on 70 pediatric IBD patients using a high throughput sequencing method for virome analysis (VirCapSeq-Vert) found that there was a very low prevalence of enteric viruses (rotavirus, calicivirus, and adenovirus) in the stools of those who were newly diagnosed with UC [78].

## 8. Experimental Models of IBD in Enteric Viruses

Murine models mimicking IBD (chemically induced IBD or transgenic mice such as IL-10 homozygous deficient mice with altered immune function) tend to simulate one aspect of IBD. These murine models can be considered useful in phases 1 and 2 of drug trials, for the testing of new biomarkers, and as experimental models for immunological and microbial studies [79,80].

The use of new cell culture systems, such as human intestinal enteroids (HIEs), opens new research opportunities for IBD because they closely mimic physiological conditions. Indeed, HIEs represent the tri-dimensional reconstruction of cellular subtypes originating from one specific tissue type [81]. Recent studies have shown that HIEs react to cellular stress and increase the dendritic cell activation and endoplasmic reticulum synthesis of proinflammatory cytokines [82]. HIEs could also be used to examine the impairment of intestinal regeneration, the disruption of the epithelial barrier during IBD flares, or gene regulation and in vivo cellular interactions [83,84,85,86]. In a secretor-dependent fashion, norovirus binding and replication could be further examined in HIEs derived from IBD tissues [87,88]. Further modeling of personalized HIEs in IBD patients will help to understand the consequences of enteric virus binding and replication at a sub-cellular level.

## 9. Enteric Viruses and Therapies Used in IBD

The control of dysbiosis is a promising therapy for reducing IBD flare-ups, as exemplified by the use of probiotics and bacteriophages to target aggressive bacteria. Such therapy could be helpful in regulating the bacterial flora, and consequently, in modulating susceptibility to enteric viruses [89]. That being said, some of the biggest advances in IBD therapy include the use of TNF-α antagonists, Janus Kinase (JAK) inhibitors, and IL-12/23 antagonists [90]. Inhibitors of lymphocyte homing have also recently been developed for use against chemokine receptor 9 (CCR9) and the α4β1 and α4β7 integrins [90]. Previous observations of the close interactions of *ATG16L* gene polymorphisms, MNV infection in Paneth cell transformation, and induction of IBD in murine models suggest that TNFα blockade may slow disease progression and help prevent intestinal necroptosis [91]. One patient diagnosed with UC who also contracted a norovirus infection showed disease improvement following administration of infliximab [92]. Furthermore, the Coxsackie–Adenovirus Receptor (CAR), which is involved in the regulation of colon cells, could decrease the TNFα-induced inflammatory response when upregulated (Figure 1) [41]. The binding of adenoviruses to inflamed colon cells will need to be further examined in the context of anti-TNF therapy. For rotavirus, it appears that the 6-thioguanine drug could inhibit virus replication through inhibition of Rac1 GTP/GDP cycling [93]. The use of 6-thioguanine, which showed limited efficacy as a biotherapy in refractory IBD, could be discussed in patients with a concomitant rotavirus infection [94].

## 10. Conclusions

Epidemiological studies, even on a large scale, are insufficient to characterize the precise role of enteric viruses in IBD relapse. IBD is influenced by microbial factors, which vary considerably between individuals, complex inflammatory pathways, and cell–virus interactions. They are also affected by confounding variables such as diet, age, smoking, or psychological stress, as demonstrated in monozygotic twins [95,96].

Real-time integrated studies are needed to decipher the mechanisms underlying IBD pathogenesis. Such studies would then contribute to the development of personalized therapeutic approaches to IBD [97,98,99]. With the advent of artificial intelligence and big data, analysis of the “integratome” (from the so-called “integrated omics”) should also open new avenues of research for enteric virus infections in IBD [100]. However, in addition to potential ethical issues (genetic analyses or biologic sampling), these types of studies remain under development and imply considerable financial costs. Nevertheless, research in this domain holds great promise for the future. Finally, vaccination against major enteric viruses (i.e., rotavirus and norovirus) could be a means of improving the management of patients suffering from IBD.

## Figures and Tables

**Table 1:** Enteric viruses and inflammatory bowel disease (epidemiological studies).

Study	Location/ Duration	Study Design	Cohort	Age Range	Samples, Materials and Methods	Conclusions Discussion
Gebhard et al., 1982 [9]	June 1978 October 1980 Minneapolis (USA)	Prospective Case control Follow-up	57 CD 20 UC 10 N	All patients: 11–64 (mean 31.5) 49 CD flare-ups 16 UC flare-ups	266 stool samples (55 during flare-up): Stool antigens -NoV and RVA Serology (3 mo follow-up)	No association 5 positive/65 flare-ups Positive serologies (NoV and RVA) correlated with severe symptoms (5/8)
Khan et al., 2009 [10]	November 2006 August 2007 Charleston (USA)	Retrospective Case control	23 CD 14 UC 9 IC 2620 N	IBD flare-ups: 11–18 (mean 15.3)	2666 stool samples Norovirus IDEIA assay kit (Dako)	Association NoV-IBD flare-ups 1119 positive 9 IBD flare-ups: CD (1/23): 1 NoV + 1 RVA UC (7/14): 7 NoV + 1 hAdV IC (0/9) Disease seasonality (*p* < 0.001)
Kolho et al., 2012 [11]	Dates unkown Helsinki (Finland)	Prospective Follow-up	18 CD 13 UC 2 IC 17 N	All IBD: 2.4–18 (mean 12.9) All non-IBD (N): 2.7–16 (mean 11)	50 stool samples RT-PCR: (NoV, RVA, hAdV, SaV, hEV)	No association IBD: 0/33 Non-IBD: 3/50 (2 NoV and 1 SaV)
Masclee et al., 2013 [12]	August 2009 November 2010 Maastricht (Netherlands)	Prospective Follow-up	170 CD 116 UC	All IBD: mean 46.2 (SD = 15.2)	286 stool samples/3-month follow-up RT-PCR (AdV, hAstV, NoV, RVA)	No association 86 IBD flare-ups RVA (2.3%), hAdV (2.3%) 200 IBD remissions RVA (0.5%), NoV (0.5%), hAdV (4.5%)
Axelrad et al., [13,14]	March 2015 May 2017 New York (USA)	Cross-sectional database analysis	277 CD 300 UC 8826 N	CD: median 37.7 UC: median 47.1 N: median 42.9	13,231 stool samples Panel PCR test (22 analytes, including hAdV, hAstV, NoV, SaV, RVA)	Association NoV-IBD flare-ups CD and UC: higher prevalence of NoV (CD; *p* < 0.05) (UC; *p* < 0.019)

CD: Crohn’s disease; UC: ulcerative colitis; N: healthy; hAdV: adenovirus; hAstV: astrovirus; hEV: enterovirus; NoV: norovirus; RVA: rotavirus; SaV: sapovirus.
